# Evaluation of Vitamin D Status in Rheumatoid Arthritis and Its Association with Disease Activity across 15 Countries: “The COMORA Study”

**DOI:** 10.1155/2017/5491676

**Published:** 2017-06-01

**Authors:** Najia Hajjaj-Hassouni, Nada Mawani, Fadoua Allali, Hanan Rkain, Kenza Hassouni, Ihsane Hmamouchi, Maxime Dougados

**Affiliations:** ^1^LIRPOS-URAC30, Faculty of Medicine and Pharmacy, Mohammed V University, Rabat, Morocco; ^2^Department of Rheumatology, El Ayachi Hospital, Ibn Sina University Hospital, Rabat-Salé, Morocco; ^3^Innovation Center, Mohammed VI University for Health Sciences, Casablanca, Morocco; ^4^Department of Physiology, Faculty of Medicine and Pharmacy, Mohammed V University, Rabat, Morocco; ^5^International School for Public Health, Mohammed VI University for Health Sciences, Casablanca, Morocco; ^6^Department of Rheumatology, Hôpital Cochin, Paris Descartes University, Assistance Publique-Hôpitaux de Paris, EULAR Center of Excellence, INSERM (U1153): Clinical Epidemiology and Biostatistics, PRES Sorbonne Paris-Cité, Paris, France

## Abstract

The aims of this study are to evaluate vitamin D status in 1413 RA patients of COMORA study from 15 countries and to analyze relationship between patients' RA characteristics and low levels of vitamin D. All demographic, clinical, and biological data and RA comorbidities were completed. The results showed that the average of vitamin D serum dosage was 27.3 ng/mL ± 15.1 [0.1–151]. Status of vitamin D was insufficient in 54.6% and deficient in 8.5% of patients. 43% of RA patients were supplemented with vitamin D and absence of supplementation on vitamin D was related to higher prevalence of vitamin D deficiency (*p* < 0.001). Finally, our study shows that the status of low levels of vitamin D is common in RA in different countries and under different latitudes. Absence of supplementation on vitamin D was related to higher prevalence of vitamin D deficiency. Low levels of vitamin D were associated with patients characteristics (age, BMI, and educational level), RA (disease activity and corticosteroid dosage), and comorbidities (lung disease and osteoporosis therapy). This suggests the need for a particular therapeutic strategy to improve vitamin D status in RA patients.

## 1. Background

In the last few years, vitamin D has been the subject of a spectacular interest and a renewed debate. Among all the topics involved, a great number of papers dealing with vitamin D relationships with autoimmune diseases have been published [1, 2]. Particularly, its role in the pathogenesis, activity, and treatment of rheumatoid arthritis (RA) has been raised, based on the observations and results of clinical and laboratory studies [1–12].

In fact, RA is an autoimmune disorder with a very complex physiopathology. The first triggering event could be the activation of antigen-dependent T cells leading to an essentially Th1 type immune response. The subsequent effects are multiple, including the activation and the proliferation of endothelial and synovial cells, recruitment and activation of proinflammatory cells, secretion of cytokines and proteases by macrophages and fibroblast-like synovial cells, and production of autoantibodies [1].

Vitamin D, as a prohormone, is considered to be able to play potential immune-suppressive roles and to exert an endocrine action on the immune system cells, generating anti-inflammatory and immunoregulatory effects [3, 4].

The rationale behind relating vitamin D and RA is based on two facts. The first one is that there is evidence indicating that patients with RA have low levels of vitamin D [5, 7, 8]. The second one is that the presence of vitamin D and VDR in macrophages, chondrocytes, and synovial cells in the joints of those patients has also been demonstrated [1].

Thus, relationship between RA and vitamin D has been extensively studied, and the results remain controversial. Several studies show that RA patients have lower levels of vitamin D [5, 6]. Either vitamin D insufficiency or deficiency was also described to be associated with higher risks of bone loss, disease activity, and disability [7–10]. However, other studies did not detect any association between RA and vitamin D concentration [12, 13].

The aims of this study are to evaluate vitamin D status in RA population of COMORA study and to analyze related factors between patients and their RA characteristics and low levels of vitamin D. We also aimed to compare vitamin D status between patients with and without vitamin D supplementation.

## 2. Patients and Methods

### 2.1. Data Source

This study is a post hoc analysis of COMORA (COMOrbidities in Rheumatoid Arthritis) cohort [14], an observational, cross-sectional, multicenter, international study. In our study, we have included patients who had a serum dosage of vitamin D among 3920 patients of COMORA study. Then 1431 were eligible to inclusion criteria.

### 2.2. Patient Recruitment

Consecutive patients visiting the participating rheumatologists were invited to enroll them in this study, only if they were at least 18years old, if patients fulfilled the 1987 American College of Rheumatology (ACR) classification criteria for RA [15], and also if patients were able to understand and complete questionnaires that were administered to them. Written informed consent was obtained from all subjects before enrollment. The scientific committee chose national principal investigators for this study. Their task was to select rheumatologists who would be representative of their country and to conduct the study in accordance with good clinical practice. The protocol was reviewed and approved by all local institutional review boards or ethics committees.

### 2.3. Methods of Data Collection and Measurements

The details of the data collected have been published previously [1].

#### 2.3.1. Patient's Characteristics

In this study, 1413 patients from 15 countries were included. Demographic characteristics and disease-specific variables (age, gender, BMI, educational level, smoking status, and disease duration) were reported. The data of drug use and vitamin D supplementation were collected. Comorbidities (hypertension, diabetes, cancer, lung, liver and renal diseases, anemia and blood diseases, hepatitis B and C, ulcer, and stomach diseases) were documented for each patient.

#### 2.3.2. Clinical Evaluation

Disease activity evaluated by DAS28 score (high > 5.1; 3.2 < moderate ≤ 5.1; 2.6 < low ≤ 3.2; and remission ≤ 2.6), functional impact of the disease evaluated by health assessment questionnaire (HAQ), and therapy (glucocorticoid intake, DMARDs, and biotherapy), and drugs affecting bone metabolism including bisphosphonates, calcium, and vitamin D supplements were described and compared according to vitamin “D” status.

#### 2.3.3. Vitamin D Status

The data of current vitamin D levels (1 ng/mL = 2.5 nmol/L) were collected. Laboratory evaluation and method of measuring serum concentration of 25-hydroxyvitamin D (25 (OH) D) were not specified in the COMORA database. It should be noted that there is no perfect consensus today on the subject of the recommended thresholds to define the limit values of serum vitamin D levels, and those that are currently recognized by a panel of experts are very variable [16–18]. In our study, vitamin D status was defined according to the Group for Research and Information on Osteoporosis (GRIO), as normal when vitamin D levels ≥ 30 ng/mL, insufficient when 10 ng/mL ≤ vitamin D levels < 30 ng/mL, and deficient when ≤ 10 ng/mL [19].

### 2.4. Statistical Analysis

Continuous variables were expressed by median ± standard deviation or median (interquartile ranges) and categorical variables as number (percentage). Comparison of categorical variables was done by chi-square or Fisher's exact test. Continuous variables were compared by Student's *t*-test. Comparison of continuous variables between normal, deficient, and insufficient vitamin D groups was done using ANOVA test. Furthermore, we used correlation analysis to compare between continuous variables. Linear regression models were performed, where factors were found to be significant to the *p* < 0.05 level in univariate analysis. Odds ratios (OR) with 95% confidence interval (95% CI) were calculated. Statistical analysis was performed using the SPSS 18.0 for Windows (SPSS 18, Chicago, IL, USA).

## 3. Results

### 3.1. Patients and Disease Characteristics

Demographic and disease characteristics of RA patients are summarized on Table 1. The average age of patients was 57.9 ± 12.8 years. High education level was received by 67.2% of patients. Most of the patients were female (83%). The median DAS28 was 3.5 ± 1.4 [0–8.2].

### 3.2. Vitamin D Status

The mean vitamin “D” serum dosage was 27.3 ng/mL ± 15.1 [0.1–151]. Status of vitamin D was normal, insufficient, and deficient in, respectively, 36.9, 54.6, and 8.5% of patients.  Table 2 illustrated vitamin D status in different countries of COMORA study.

### 3.3. Patients' Comorbidities

Hypertension, anemia, blood diseases, ulcer, and stomach diseases were the most common comorbidities in our study (Table 1). Patients with vitamin D insufficiency had more comorbidities and were significantly associated with lung diseases and osteoporosis therapy (*p* = 0.006 and *p* = 0.04, resp.) (Table 4).

### 3.4. Association Analysis between Vitamin D Status and Patients RA Characteristics

In univariate analysis (Tables 3 and 4), low levels of vitamin D were associated with higher age (*p* < 0.001), higher BMI (*p* = 0.02), higher disease activity (*p* = 0.01), more current daily dose of corticosteroids (*p* = 0.04), lower level of education (*p* < 0.001), antiosteoporotic treatment (*p* = 0.04), and presence of lung diseases (*p* = 0.006). The vitamin D levels were inversely correlated with disease activity assessed by DAS28 (*r* = −0.104; *p* < 0.001).

In multivariate analysis, all those variables except presence of lung diseases remain significantly associated with a bad status of vitamin D. Association analysis between vitamin D status and patients RA characteristics is summarized in Table 5.

### 3.5. Relationship between Vitamin D Supplementation and Vitamin D Status

In this cohort, 43% of RA patients were supplemented with vitamin D (Table 6). Comparing vitamin D status between patients with and without vitamin D supplementation showed that deficiency on vitamin D was more prevalent in patients without vitamin D supplementation (*p* < 0.001). Prevalence of a normal status of vitamin D was significantly higher in patients with vitamin D supplementation (*p* < 0.001).  Table 7 illustrated those findings.

## 4. Discussion

This study showed that prevalence of low vitamin D levels is common in RA patients. This prevalence was really varied between countries, with the highest rates found in Korea and the lowest rates in Italy (71% and 36%, resp.). This variability may be explained by some differences among the populations, such as racial/ethnic differences, regional climates, lack of sun exposure, veiled clothing style, or latitude [7, 20, 21]. Indeed we have long considered that vitamin D insufficiency and deficiency were the preserve of the few sunny countries, particularly in Northern Europe, with a North-South gradient in Europe. In fact, recent data shows that the tropicals or subtropicals, such as Central America and the Middle East, particularly Asia, are also widely affected [22]. This has several explanations: no sun exposure in hot countries, coloured skin which synthesizes less vitamin D, and wearing protective clothing. This variability could also be related to the well-known vitamin D receptor (VDR) gene polymorphism and its expression across different populations, able to influence RA severity [20, 23, 24].

Current epidemiological data indicates a high prevalence of vitamin D insufficiency in patients with autoimmune diseases and especially in RA when compared to the general population [25, 26]. However according to some studies, there is a great variability most likely because of the size and the methodology used in these studies or according to the characteristics of the populations. In Turkey and India, the prevalence of vitamin D insufficiency was, respectively, about 68% and 90% [20, 27]. In Denmark, Haga et al. evaluated it up to 33.4% [28]. This prevalence was around 43% in an Italian cohort [8]. In the USA, the prevalence ranges from 41% up to 84% [5, 29], and it was common in African Americans with recent-onset RA, affecting approximately half of this population [7]. When considering countries latitudes, some studies have shown that vitamin D insufficiency, together with a higher prevalence of RA, was more common among Northern patients than in Southern European patients [21]. Even in northern latitudes, vitamin D insufficiency has been linked to higher latitude as it further characterizes Greenlanders versus Danes [30].

Although the primary determinant of vitamin D status is sunlight exposure [31], the prevalence of vitamin D deficiency is paradoxically higher in sunny countries [31]. In Morocco,* in spite of the sunny climate*, prevalence of vitamin D insufficiency has been demonstrated as exceeding that of the European cohorts, as it affects about 91% of Moroccan women [32, 33].

One hypothesis for vitamin D insufficiency throughout the countries may be latitude. Indeed it has been described that prevalence of RA increases with higher latitude [34, 35]. However, it has been described that vitamin D levels are low in RA patients in low latitude [9, 19], especially in above latitude 33° (north of a line through the North of Morocco, Northern Algeria, Iran, Iraq, Japan, and Los Angeles). It was considered that it is not possible to synthesize vitamin D by exposure to sunlight for some winter months [19]. Unfortunately, in our study, this status of latitude could not be studied to verify this relationship.

In this study, vitamin D status was associated with several independent factors that have previously been shown to affect vitamin D [23]. We found a significant association with low levels of vitamin D and age, educational level, and BMI. This correlation remained significant even after multivariate adjustment (Table 6). In fact, regardless of the disease, the dietary intake and cutaneous production of vitamin D decreases with age and adiposity [35, 36]. Also patients, who had a high educational level, had better vitamin D status, with a higher prevalence of normal vitamin D level. This is probably due to their better understanding of their disease and its complications, as well as their greater ability to perform the dosage of vitamin D.

There was a negative relationship between vitamin D levels and RA disease activity in our study. After multivariate adjustment, this correlation between vitamin D levels and DAS28 scores persisted. That is to say, DAS28 scores increase when vitamin D levels decrease.

In fact, the association between low vitamin D levels and active RA is widely debated in the literature with conflicting data. Our results are consistent with some studies [5, 8–10, 12, 21, 29, 37, 38], while others did not find any significant relationship between RA and low vitamin D levels [7, 11, 39, 40]. This relationship may be related to latitude as it was described. In low-latitude regions, there is not a lot of sun exposure because of the months of rain, which could exacerbate joint pain and increase the DAS28 scores [9].

Osteoporosis, whose prevalence was estimated by percentage of patients taking a specific antiosteoporotic drug in our study, was significantly higher in patients with low vitamin D levels. This is probably related to RA which generates bone loss and corticosteroids use which increases bone remodeling and decreases bone density. This can also be explained by the fact that a low vitamin D level is involved in the pathogenesis of osteoporosis [15, 41, 42]. Indeed, it has been shown that low vitamin D levels may be the result of changes in cortical bone and lead to bone fragility [22].

In our study, vitamin D insufficiency was also significantly associated with other comorbidities in RA mostly lung diseases. In fact, it was described that patients with respiratory disease are frequently deficient in vitamin D [43]. This is probably related to the fact that respiratory monocytes/macrophages and epithelial cells constitutively express the vitamin D receptor. Smoking also may lead to the deficiency of vitamin D, but in our study, there was no significant association between smoking and vitamin D status. Other studies have shown that lower vitamin D levels increase the VDR gene mutations. This could be an explanation of increase risk of lung cancer [44, 45]. This was not found in our study.

Glucocorticoids intake remains independently and inversely associated with low vitamin D levels in our study. The GRIO had already recommended a systematic supplementation in patients using a long-term corticotherapy [19]. Recently, corticosteroids and vitamin “D” have been subjects to experimental studies and clinical trials in a plethora of autoimmune diseases. These molecules are ligands for the nuclear receptor, which results in a direct regulation of transcription of genes involved in the immune system. This eventually leads to an immune response less proinflammatory and, therefore, beneficial in autoimmune diseases [46].

In our study, 43% of the patients were supplemented with vitamin D. This prevalence varied widely between countries. This is could be explained by the variability in the number of patients included in each country. Patients, who were supplemented by vitamin D in our study, had a better vitamin D status than those who had no vitamin D supplementation. And the opposite, patients who were not supplied had a vitamin D deficiency. This implies necessity to optimize doses of vitamin D supplementation in RA patients. Some studies have concluded that clinical improvement in RA patients treated with vitamin D was strongly correlated with the immunomodulating potential of vitamin D administration [3, 47], while others suggest that antirheumatic drugs combined with vitamin D should be recommended for RA patients, in order to prevent and treat osteoporosis and improve its effect on disease activity [46].

Our study had some limitations. Firstly, our data included 1413 RA patients that are corresponding to 36% of the COMORA study population. This is reflecting the rheumatologist practice in real life regarding prescription of vitamin D dosage in RA patients. This lack of vitamin D dosage might be related to the difference in attitudes of health practitioners on the request of the dosage of vitamin D or to the cost of this assay and its repayment by the health insurance in some countries.

Secondly, the method of vitamin D dosing was not specified in the COMORA database. Certainly, there is variability in methods of assay in each country. In fact there are several ways to measure 25 (OH) D (radio-immunoassays, enzyme-linked assays, and liquid chromatography with mass spectrometry). The precision and accuracy of the assays especially in nonreference laboratories remain problematic. There is also no reference method or international standard today for the determination of the 25 (OH) vitamin D. The characteristics required to define future reference method, which will probably be mass spectrometry after chromatography gas or liquid, have been proposed recently [48].

Thirdly, the association between vitamin D status and osteoporosis in our study is to some extent unsatisfying. In fact, the prevalence of osteoporosis has been estimated on the basis of percentage of patients taking antiosteoporotic drugs. We have certainly omitted patients with osteoporosis without any antiosteoporotic treatment.

Finally, schemes of vitamin D supplementation were not available in COMORA database. Consequently, it is not clear at all if those schemes were really adequate or not.

Despite those limitations, our data has several strengths. Comparing to other studies focusing on vitamin D status and RA, our data has the advantage to include a large number of patients. We have described vitamin D in 15 countries. Prevalence of low vitamin D levels seems to be high in most of them. Nevertheless, we are not able to make any adequate comparison on vitamin D status between the 15 countries as well. This is due to discrepancies in population number, age, and other RA and disease characteristics. In addition to this, our study showed that status on vitamin D was better in patients who have vitamin D supplementation.

## 5. Conclusion

A status of low level of vitamin D is common in RA in different countries and under different latitudes. Absence of supplementation on vitamin D is related to higher prevalence of vitamin D deficiency. Many factors related to patients and their diseases seem to be associated with low level of vitamin D.

Vitamin D supplementation by active natural or synthetic analogs could possibly be used as an adjunct therapy with DMARDs in patients with active RA. Moreover, it can be considered as an inexpensive, potentially safe, and beneficial adjuvant aimed to reducing disease severity as well as joint and bone destruction. Nevertheless, randomized and controlled trials are necessary to determine from which reference levels deficiency or insufficiency should be considered. It is also necessary to evaluate which mechanism and which dosing of drug to choose. All this is to achieve pharmacological and clinical efficacy and safety, suggesting specific treatment strategies especially in RA patients.

## Figures and Tables

**Table 1:** Demographic and disease characteristics of rheumatoid arthritis patients.

Characteristics	*N* = 1413
Age (years)^¹^	57.9 ± 12.8 [18–88]
Female sex^2^	1172 (82.9)
High educational level^2^	
(≥secondary)	950 (67.2)
BMI (kg/m^2^)^¹^	26.3 ± 5 [15–53.1]
Current/past smoking^2^	214 (15.1)
Disease duration (years)^3^	8.3 (3.6–15.2)
DAS 28^¹^	3.5 ± 1.4 [0–8.2]
Disease activity	
High^2^	231 (16.3)
Moderate^2^	576 (40.8)
Low^2^	208 (14.7)
Remission^2^	398 (28.2)
Vitamin D level^2^	27.3 ± 15.1 [0.1–151]
HAQ^2^	1 ± 0.6 [0.4–1]
Glucocortocoid therapy intake^2^	691 (48.9)
Current dose of glucocortocoid (mg/day)^3^	0 [0–5]
DMARDs^2^	
Immunosuppressor	17 (1.2)
APS	238 (16.8)
Salazopyrin	70 (4.9)
Golds	57 (4)
Methotrexate	763 (54)
Biotherapy^2^	173 (12.2)
Comorbidities^2^	
Hypertension	494 (35)
Diabetes	133 (9.4)
Cancer	42 (3)
Lung diseases	110 (7.8)
Renal diseases	40 (2.8)
Anemia and blood diseases	165 (11.8)
Hepatitis B	39 (2.8)
Hepatitis C	18 (1.3)
Liver diseases	51 (3.6)
Ulcer and stomach diseases	166 (11.8)

^¹^Mean ± DS; ^2^number and percentage; ^3^median; BMI: body mass index; DAS28: Disease Activity Score using 28 joints; HAQ: health assessment questionnaire; APS: antimalarial drug, DMARDs: Disease-Modifying Antirheumatic Drugs.

**Table 2:** Vitamin D status according to countries.

	*N* (%)^*∗*^	Vitamin D normal^1^ *N* (%)^*∗*^	Vitamin D insufficiency^2^ *N* (%)^*∗*^	Vitamin D deficiency^3^ *N* (%)^*∗*^
Argentina	120 (100)	33 (27.5)	71 (59.2)	16 (13.3)
Austria	120 (100)	37 (30.8)	68 (56.7)	15 (12.5)
Egypt	1 (100)	0 (0)	1 (100)	0 ( 0)
France	309 (100)	146 (47.2)	152 (49.2)	11 ( 3.6)
Germany	45 (100)	13 (28.9)	30 (66.7)	2 (4.4)
Hungary	87 (100)	25 (28.7)	53 (60.9)	9 (10.3)
Italy	81 (100)	46 (56.8)	29 (35.8)	6 (7.4)
Korea	111 (100)	23 (20.7)	79 (71.2)	9 (8.1)
Morocco	34 (100)	8 (23.5)	20 (58.8)	6 (17.6)
Netherlands	3 (100)	0 (0)	2 (66.7)	1 (33.3)
Spain	133 (100)	37 (27.8)	83 (62.4)	13 ( 9.8)
Taiwan	88 (100)	7 (8.0)	58 (65.9)	23 (26.1)
UK	1 (100)	0 (0)	1 (100)	0 (0)
Uruguay	9 (100)	3 (33.3)	5 (55.6)	1 (11.1)
USA	271 (100)	143 (52.8)	120 (44.3)	8 ( 3)
^*∗*^Total	1413 (100)	521 (36.9)	772 (54.6)	120 (8.5)

^*∗*^Number and percentage; ^1^vitamin D ≥ 30 ng/mL, ^2^10 ng/mL ≤ Vitamin D < 30 ng/mL, and ^3^vitamin D < 10 ng/mL.

**Table 3:** Relationship between demographic and disease characteristics of RA patients and vitamin D status.

	Vitamin D normal^*∗*^	Vitamin D insufficiency^*∗∗*^	Vitamin D deficiency^*∗∗∗*^	*p*
Age (years)^1^	57.2 ± 12.7 [19–86]	56.4 ± 12.8 [18–88]	59.2 ± 12.8 [19–88]	**<0.001**
Female sex^2^	426 (36.3)	640 (54.6)	106 (9)	0.27
BMI (kg/m)^1^	26.9 ± 5.7 [15.1–53.9]	26.4 ± 5.7 [15.6–50.2]	25.9 ± 5.2 [16.1–47.2]	**0.02**
Smoking current/past^2^	204 (35.8)	152 (26.7)	214 (37.5)	0.8
High educational level^2^	377 (39.7)	509 (53.6)	64 (6.7)	**<0.001**
Disease duration (years)^1^	11.8 ± 10 [0.02–56.2]	10.1 ± [0.02–56.2]	10.3 ± 9.2 [0–54]	1.0
Glucocorticoid intake^2^	249 (36)	208 (30.1)	234 (33.9)	**0.04**
DAS28^1^	3.3 ± 0.06 [0–8.2]	3.5 ± 0.05 [0.4–8.1]	3.6 ± 0.1 [1.1–7.3]	**0.01**
HAQ^1^	0.9 ± 0.6 [0.4–3]	1.02 ± 0.6 [0.4–3]	1.02 ± 0.6 [0.4–2.9]	0.5

^1^Mean ± DS; ^2^number and percentage; ^*∗*^vitamin D ≥ 30 ng/mL, ^*∗∗*^10 ng/mL ≤ vitamin D < 30 ng/mL, and ^*∗∗∗*^vitamin D < 10 ng/mL; BMI: body mass index; DAS28: Disease Activity Score using 28 joints; HAQ: health assessment questionnaire; *p* significant if ≤0.05.

**Table 4:** Association analysis between comorbidities and vitamin D status.

Comorbidities^*∗*^	Vitamin D normal^1^	Vitamin D insufficiency^2^	Vitamin D deficiency^3^	*p*
Hypertension	181 (36.6)	272 (55.1)	41 (8.3)	0.9
Diabetes	46 (34.6)	76 (57.1)	11 (8.3)	0.8
Dyslipidemia	41 (33.6)	66 (54.1)	15 (12.3)	0.2
Renal deficiency	15 (37.5)	21 (52.5)	4 (10)	0.9
Anemia and blood diseases	66 (40)	88 (53.3)	11 (6.7)	0.5
Cancer	18 (42.9)	21 (50)	3 (7.1)	0.7
Osteoporosis therapy	124 (42.6)	150 (51.5)	17 (5.8)	** 0.04**
Lung diseases (COPD, asthma, cancer)	50 (45.5)	45 (40.9)	15 (13.6)	** 0.006**
Liver diseases	20 (39.2)	26 (51)	5 (9.8)	0.8
Ulcer and stomach disease	156 (33.7)	91 (54.8)	19 (11.4)	0.3

^*∗*^Number and percentage; ^1^vitamin D ≥ 30 ng/mL, ^2^10 ng/mL ≤ vitamin D < 30 ng/mL, and ^3^vitamin D < 10 ng/mL; *p* significant if ≤0.05.

**Table 5:** Multivariate analysis between vitamin D levels and characteristics of RA patients.

	*β*	IC	*p*
Age (years)	0.14	[0.10; 0.23]	**<0.001**
BMI (kg/m^2^)	−0.07	[−0.35; −0.06]	** 0.004**
High educational level	0.12	[2.39; 5.85]	**<0.001**
DAS28	−0.05	[−1.21; −0.05]	**0.03**
Glucocorticoid intake (mg/day)	−0.08	[−0.30; −0.06]	** 0.003**
Osteoporotic therapy intake	−0.07	[−4.53; −0.65]	**0.009**
Lung diseases	−0.01	[−4.82; −2.74]	0.58

*p* significant ≤ 0.05.

**Table 6:** Vitamin D intake according to countries.

Country^*∗*^	Patients with vitamin D supplementation	Patients without vitamin D supplementation	Total
Argentina	72 (60)	48 (40)	120 (100)
Austria	37 (30.8)	83 (69.2)	120 (100)
Egypt	0 (0)	1 (100)	1 (100)
France	99 (32)	210 (68)	309 (100)
Germany	21 (46.7)	24 (53.3)	45 (100)
Hungary	46 (52.9)	41 (47.1)	87 (100)
Italy	44 (54.3)	37 (45.7)	81 (100)
Korea	58 (52.3)	53 (47.7)	111 (100)
Morocco	12 (35.5)	22 (64.7)	34 (100)
Netherlands	2 (66.7)	1 (33.3)	3 (100)
Spain	57 (42.9)	76 (57.1)	133 (100)
Taiwan	64 (72.7)	24 (27.3)	88 (100)
UK	1 (100)	0 (0)	1 (100)
Uruguay	1 (11.1)	8 (88.9)	9 (100)
USA	94 (34.7)	177 (65.3)	271 (100)
Total	608 (43)	805 (57)	1413 (100)

^*∗*^Number and percentage.

**Table 7:** Vitamin D status according to vitamin D supplementation.

	Patients with vitamin D supplement^*∗*^ (*n* = 805)	Patients without vitamin D supplement^*∗*^ (*n* = 608)	*p* value
Vitamin D normal^1^ (*N* = 521)	353 (43.9)	168 (27.6)	**<0.001**
Vitamin D insufficiency^2^ (*N* = 772)	403 (50.1)	369 (60.7)	**<0.001**
Vitamin D deficiency^3^ (*N* = 120)	49 (6.1)	71 (11.7)	**<0.001**

^*∗*^Number and percentage; ^1^vitamin D ≥ 30 ng/mL, ^2^10 ng/mL ≤ vitamin D < 30 ng/mL, and ^3^vitamin D < 10 ng/mL; *p* significant ≤ 0.05.

## Abbreviations

ACR: American College of Rheumatology
APS: Antimalarial drug
BMI: Body mass index
CRP: Creative reactive protein
CI: Confidence interval
DAS28: Disease Activity Score using 28 joints
DMARDs: Disease-Modifying Antirheumatic Drugs
ESR: Erythrocyte sedimentation rate
GRIO: Group for Research and Information on Osteoporosis
HAQ: Health assessment questionnaire
SD: Standard deviation
SJC: Swollen joint count
TJC: Tender joint count
PhGA: Physician global assessment
RA: Rheumatoid arthritis
UVR: Ultraviolet ray
VDR: Vitamin D receptor.

## Data Access

The database is available from Pr. Najia Hajjaj-Hassouni.
